# Serological and spatial analysis of alphavirus and flavivirus prevalence and risk factors in a rural community in western Kenya

**DOI:** 10.1371/journal.pntd.0005998

**Published:** 2017-10-17

**Authors:** Elysse N. Grossi-Soyster, Elizabeth A. J. Cook, William A. de Glanville, Lian F. Thomas, Amy R. Krystosik, Justin Lee, C. Njeri Wamae, Samuel Kariuki, Eric M. Fèvre, A. Desiree LaBeaud

**Affiliations:** 1 Departments of Pediatrics, Infectious Disease Division, Stanford University School of Medicine, Stanford, California, United States of America; 2 Zoonotic and Emerging Diseases Group, International Livestock Research Institute, Nairobi, Kenya; 3 Centre for Immunity, Infection and Evolution, Institute for Immunology and Infection Research, School of Biological Sciences, University of Edinburgh, Ashworth Laboratories, Edinburgh, United Kingdom; 4 Quantitative Sciences Unit, Stanford University School of Medicine, Stanford, California, United States of America; 5 Department of Microbiology, School of Medicine, Mount Kenya University, Thika, Kenya; 6 Centre for Microbiology Research, Kenya Medical Research Institute (KEMRI), Nairobi, Kenya; 7 Institute of Infection and Global Health, University of Liverpool, Leahurst Campus, Neston, United Kingdom; Florida Department of Health, UNITED STATES

## Abstract

Alphaviruses, such as chikungunya virus, and flaviviruses, such as dengue virus, are (re)-emerging arboviruses that are endemic in tropical environments. In Africa, arbovirus infections are often undiagnosed and unreported, with febrile illnesses often assumed to be malaria. This cross-sectional study aimed to characterize the seroprevalence of alphaviruses and flaviviruses among children (ages 5–14, n = 250) and adults (ages 15 ≥ 75, n = 250) in western Kenya. Risk factors for seropositivity were explored using Lasso regression. Overall, 67% of participants showed alphavirus seropositivity (CI_95_ 63%–70%), and 1.6% of participants showed flavivirus seropositivity (CI_95_ 0.7%–3%). Children aged 10–14 were more likely to be seropositive to an alphavirus than adults (p < 0.001), suggesting a recent transmission period. Alphavirus and flavivirus seropositivity was detected in the youngest participants (age 5–9), providing evidence of inter-epidemic transmission. Demographic variables that were significantly different amongst those with previous infection versus those without infection included age, education level, and occupation. Behavioral and environmental variables significantly different amongst those in with previous infection to those without infection included taking animals for grazing, fishing, and recent village flooding. Experience of recent fever was also found to be a significant indicator of infection (p = 0.027). These results confirm alphavirus and flavivirus exposure in western Kenya, while illustrating significantly higher alphavirus transmission compared to previous studies.

## Author summary

There are many examples of recent emergence of mosquito-borne viruses, such as chikungunya virus outbreaks throughout the Caribbean in 2013, Zika virus outbreaks throughout Southern and Central America in 2015, and yellow fever virus in Brazil in 2017. Each outbreak draws attention to the limits associated with predicting future outbreaks. This study expands our understanding of risk factors for exposure to two common genera of mosquito-borne viruses, alphaviruses and flaviviruses. Risk factors identified include simple demographic factors, such as age or sex, and behaviors associated with occupation or livelihood around the home. Behaviors enhancing or limiting contact with mosquitoes are also significant predictors, as mosquitoes drive transmission. More clearly defining the epidemiology of these infections within a population can elevate the accuracy and efficacy of public health initiatives that fuel community education and awareness, and outbreak prediction and monitoring can be elevated to a new level of accuracy and efficacy.

## Introduction

Arthropod-borne viruses (arboviruses), such as the alphavirus chikungunya (CHIKV), and the flavivirus dengue (DENV), represent a multi-dimensional, ongoing threat for current and future generations[1–6]. Sudden and pervasive outbreaks have become an increasingly regular occurrence over the last decade, illustrating the intensity at which arboviruses can spread and affect naïve populations[1, 7]. Many alphaviruses and flaviviruses are primarily transmitted by the same vector, the *Aedes aegypti* mosquito[8, 9], which is found in most regions of Kenya, in both rural and urban sites [10–12]. Due to the shared primary vector species, DENV and CHIKV are now co-endemic in many regions of the world, including Asia, Africa, South and Central America, and the Caribbean[13–15].

Acute symptoms of many alphavirus and flavivirus infections are generally representative of nonspecific and mild febrile disease, with the addition of a possible rash, arthralgia, and arthritis[16–18]. For this reason, accurate differential diagnosis is necessary for determining appropriate symptom-specific treatment, and avoiding non-specific clinical diagnoses that often lead to inappropriate treatments, most commonly those used for malaria[19].

Many previous studies describing virus-specific prevalence in African countries report conflicting results[20–23]. This may be due to regional distribution of vectors [12, 24], seasonal fluctuations in climate and flooding, parallel sylvatic transmission cycles, demographics associated with previous exposure and acquired immunity, and other factors involved in aiding viral spread[25]. In this study, we aimed to increase the knowledge regarding seroprevalence and factors associated with increased exposure to alphaviruses and flaviviruses in a population of children and adults living in western Kenya.

## Materials and methods

### Study area

The study area spans an approximately 3,200 Km^2^ semi-circle centered in the town of Busia[26]. This area is largely representative of the wider Lake Victoria Crescent ecosystem, which includes regions in Kenya, Uganda, and Tanzania. The study area is a rural area of approximately 1.4 million people [27], with the majority of people involved in mixed farming of crops and livestock[26].

### Sample population

Serological samples and demographic data were collected by weighted and stratified random sampling of 416 homesteads between August 2010 and July 2012 as part of a cross-sectional study of zoonotic infections in western Kenya[26, 28–30]. Sera were analyzed to determine alpha- and flavivirus seroprevalence among children and adults. Random sampling of homesteads within the original study was stratified within sub-locations, the smallest administrative unit in Kenya. The number of homesteads to select per sub-location (between 1 and 8) was proportional to the expected cattle population, so that more homesteads were sampled in sub-locations with larger cattle populations. A household was defined as all people identified by the head of household as being an occupant at the time of recruitment, to the extent that food is regularly shared from the household pot within the past 4 weeks. GPS coordinates were obtained for every homestead in the study using a handheld Garmin GPS unit. A maximum of 25ml of venous blood was collected for the original study. Biobanked aliquots of serum stored at -80°C were used for this study.

Individuals aged 5 years or older from consenting homesteads were included in our study population. A subset of 250 samples per age category (adults (ages ranging from 15 to ≥ 75 years) and children (ages ranging from 5 to 14 years)) was selected as a representative sampling from the original cohort of 2,106 subjects (879 adults and 1,227 children). The 250 samples from adults and children were selected from a randomized sorting of the original study samples in Excel. Relevant demographic and health information was collected from questionnaire data regarding health and vaccine history, behavioral lifestyle and practices within the homestead and were linked to seropositivity. The original data pertaining to this study are available in an online repository. Exclusion criteria included subjects with severe anemia and those in their third trimester of pregnancy.

### Serological analysis

Sera were tested by indirect ELISA for the presence of anti-CHIKV and anti-DENV IgG antibodies, as described previously[2, 31]. Nunc-immuno 96-well plates were coated with CHIKV antigen (derived from the 181/25 vaccine vector strain, and supplied by Dr. Mark Heise, University of North Carolina School of Medicine, Chapel Hill, NC 27599), in a carbonate coating buffer, or DENV_1–4_ antigen (derived from four serotypes[32–34]: DENV1, Western Pacific 74; DENV2, S16803; DENV3, CH53489; and DENV4, TVP360, and provided by Dr. Eva Harris, University of California Berkeley, Berkeley, CA 94720) in a PBS/0.01% NaN_3_ buffer (1:400, 50μl/well). Plates were coated overnight at 4°C. Plates were washed with a PBS/0.01% Tween-20/0.01% NaN_3_ wash buffer, blocked with blocking buffer (5% powdered milk in PBS) for two hours at 37°C, and washed prior to adding the samples. Serum samples (1:200 in blocking buffer) were added in duplicate (50μl/well) and incubated overnight at 4°C. Plates were washed, coated with goat anti-human IgG conjugated to alkaline phosphatase (AP) (1:1000 in blocking buffer, 50μl/well), and incubated for one hour at 37°C. Following incubation, plates were washed and AP substrate in PnPP buffer (1ug/1ml) was added (100μl/well). After a 30-minute incubation at 37°C, optical density was read at 405nm. Cut-off values for seropositive and seronegative results were determined against plaque-reduction neutralization tests (PRNT)-confirmed serum samples for CHIKV and DENV, by calculating three times the negative control, and at least half of the positive control reading.

### Data analysis

All statistical analysis was conducted using *R* programming language open-source software[35]. Prevalence was calculated by determining the percentage of serologically positive samples within each age category. Bivariate analysis of each of the potential predictors of either alphavirus or flavivirus exposure was performed using the Chi-square or Fisher’s Exact test. Demography and health data derived from the participant questionnaires were analyzed using LASSO (least absolute shrinkage and selection operator) generalized linear mixed models regression (the *glmmLasso* package)[36] to determine factors associated with increased risk of alphavirus and flavivirus seropositivity. The glmmLasso package was used because it allows the inclusion of a random effect (household) in the LASSO variable selection process. With many potential predictors of exposure available in this study and limited prior knowledge of which are definitively predictive of DENV/CHIKV infection, the LASSO procedure shrinks unimportant variable estimates to 0 and allows selection of the most correlated variables based on this dataset. The LASSO procedure is typically used when sample size is relatively small when compared to the number of variables of interest. Akaike’s Information Criteria (AIC) and Bayesian Information Criteria (BIC) were used to run iterations of glmmLasso to identify statistically predictive variables for alphavirus and flavivirus infection. The optimal lambda tuning parameter was chosen by the minimum AIC value. A cross-validation (CV) method was also used to run iterations of glmmLasso to identify variables significantly related to the increased odds of alphavirus and flavivirus exposure. Models produced using CV are created using “train” datasets and the resulting optimal model is validated using “test” datasets. Variables included in the glmmLasso model were selected based on their relevance to arbovirus and vector exposure, including basic demographic data such as age group, sex, community, education level, and occupation; proxies for time spent outdoors such as frequency of hunting, fishing, and grazing behaviors; and animal contact such as whether livestock and poultry have access to the homestead buildings; whether wildlife, including rats, were observed; village flooding and drought history; water sources used during wet and dry seasons; and health-related data such as recent illness, smoking behavior, and vaccination history. All variables were subject to a random effect of homestead, defined by shared exact northing and easting GPS coordinates.

Kernel density analysis was used to map the distribution of CHIKV and DENV seropositive events within the geographic limits of the study region[37]. Samples that tested seropositive for previous alphavirus or flavivirus exposure were mapped within the study area based on GPS coordinates collected at the time of sample collection. Maps were created using ArcGIS software by ESRI (ESRI ArcGIS Desktop: release 10.3.1; Redlands, CA). For each case, case level data was projected using Arc 1960 Universal Trans Mercator Zone 37s (21037) and geographic coordinate system GCS_Arc_1960. Kernel density analysis was performed using the geoprocessing tools within ArcGIS 10.3.1 (ESRI) using a bandwidth of 9,000 meters based on incremental spatial autocorrelation analysis to select a distance band reflecting maximum spatial autocorrelation. Various methods are suggested for selecting a bandwidth based on the biological bases of underlying disease mechanisms and quantitative criteria[38]. Human movement also impacts transmission patterns[39], and estimates of the biological distance at which dengue and chikungunya transmission occurs vary widely[40–43]. Resulting kernel density raster files were contoured and the top 50% of contours selected and mapped.

Spatial scan statistics were performed using SatScan[44] with Bernouli distribution [45]. All data points were separated by infection status for cases versus controls for each DENV and CHIKV. SatScan searched for high clusters using a maximum spatial cluster size of 50 percent of population at risk and a circular window shape. Secondary clusters were only reported without geographic overlap. Resulting clusters were mapped using ArcGIS software by ESRI (ESRI ArcGIS Desktop: release 10.3.1; Redlands, CA). Maps utilized for kernel density and spatial scan statistics were produced using basemaps OpenStreetMap Data from mapbox (https://www.mapbox.com/), an open source mapping resource.

Bodies of water in the study area were mapped, and a near table was generated by calculating the shortest path based on a spheroid (geodesic) to the nearest body of water using the ArcGIS geoprocessing toolbox. Median distance to nearest water body was compared across infected and non-infected subjects using the Wilcoxon rank-sum test.

### Ethics statement

Serological samples and demographic data were collected by weighted and stratified random sampling of 416 homesteads between August 2010 and July 2012 as part of a cross-sectional study of zoonotic infections in western Kenya. Ethical approval for study was granted by the Kenya Medical Research Institute (KEMRI) Ethical Review Board (SC1701); participants of the original study consented to long term storage and further use of their anonymized samples. Subsequent analysis of biological material as described here was approved by the Stanford University Institutional Review Board (R01: IRB-31488); all participants and/or legal guardians provided written informed consent.

## Results

Of the 500 samples tested, 66.9% (n = 335, CI_95_ 62.7%–70.9%) of all participants were seropositive for previous alphavirus exposure, as indicated by the presence of anti-CHIKV IgG, and 1.6% (n = 8, CI_95_ 0.8%–3.1%) were seropositive for previous flavivirus exposure, indicated by the detection of anti-DENV IgG. Comparatively, adults (age groups 15–24 through 75+) had a higher rate of alphavirus seropositivity (78.7%) than children (age group 5–14) (42%). Flavivirus seropositives were only identified in individuals under the age of 45, with the highest percentage (5.2%) of positives in individuals aged 15–24 years (n = 76). Only 1 of the 2 children seropositive for flavivirus IgG was also seropositive for alphavirus IgG, whereas all six of the adults that were seropositive for flavivirus IgG were also seropositive for alphavirus IgG. Of the flavivirus seropositives (n = 8), 62.5% (n = 5) had not been vaccinated against yellow fever, indicating that false positives were not a concern for the vaccinated population. History of vaccination against yellow fever was not significantly correlated with alphavirus infection (p = 0.06). Although this may suggest weak evidence of an inverse relationship between vaccination against yellow fever and flavivirus infection, the yellow fever vaccination variable was not selected as a significant variable by further multivariable modeling.

Biological sex was not a statistically significant factor for alphavirus or flavivirus seroprevalence in adults (p = 0.16). Seroprevalence for alphaviruses in children was nearly equal for female (n = 70 (57.4%)), and male (male: n = 71 (55%)) participants. Two female children tested positive for anti-DENV IgG, resulting in 0.8% (CI_95_ 0.1%–2.9%) seroprevalence for flaviviruses in females. No flavivirus positive cases were identified in male child participants.

Using Chi-square test and fisher’s exact test, a range of variables was assessed for their influence on the risk of alphavirus or flavivirus infection (Table 1). Given the low number of flavivirus positives, statistical analysis described in Table 1 details infection, inclusive of either alphavirus or flavivirus positives, compared to no infection, inclusive of all seronegatives. Occupation type between ‘infection’ and ‘no infection’ groups was significantly different (p < 0.001). Variables relating to keeping livestock, including feeding and sources of water for livestock in wet and dry seasons, husbandry practices, meat and dairy consumption, and slaughtering practices were not indicators of previous alpha- or flavivirus infection, with the exception of grazing in the last 12 months (p < 0.001). Proximity of wildlife to the home was correlated with infection (p = 0.05). Recent flooding was also significantly correlated with previous alpha- or flavivirus infection (p < 0.001), whereas drought was not significant (p = 0.628). Fishing in the last 12 months was also found to be significant (p = 0.018). Water collection during dry seasons was not significant, regardless of water source, yet obtaining water from a well during the wet season was found to correlate with infection (p = 0.05).

**Table 1:** Descriptive characteristics associated with arbovirus infection.

		Alphavirus (n = 335) or Flavivirus (n = 8) infection		
Characteristics	Total Population (n = 499 (%))	No Infection (n = 161 (%))	Infection (n = 338 (%))	p-value
Age Group–n (%)				<0.001*
05–14	250 (50.1)	108 (67.1)	142 (42.0)	
15–24	76 (15.2)	19 (11.8)	57 (16.9)	
25–34	52 (10.4)	15 (9.3)	37 (10.9)	
35–44	42 (8.4)	3 (1.9)	39 (11.5)	
45–54	37 (7.4)	8 (5.0)	29 (8.6)	
55–64	19 (3.8)	3 (1.9)	16 (4.7)	
65–74	21 (4.2)	5 (3.1)	16 (4.7)	
75+	2 (0.4)	0 (0.0)	2 (0.6)	
Sex–n (%)				
Male	239 (47.9)	85 (52.8)	154 (45.6)	0.16
Community–n (%)				0.46
Luhya	254 (50.9)	83 (51.6)	171 (50.6)	
Luo	106 (21.2)	29 (18.0)	77 (22.8)	
Saboat	1 (0.2)	0 (0.0)	1 (0.3)	
Samia	68 (13.6)	21 (13.0)	47 (13.9)	
Teso	70 (14.0)	28 (17.4)	42 (12.4)	
Education Level–n (%)				<0.001*
None	39 (7.8)	10 (6.2)	29 (8.6)	
Pre-school	49 (9.8)	30 (18.6)	19 (5.6)	
Primary	358 (71.7)	104 (64.6)	254 (75.4)	
Secondary and above	52 (10.4)	17 (10.6)	35 (10.4)	
Occupation–n (%)				<0.001*
Farmer	145 (29.1)	24 (16.9)	121 (38.1)	
Student	244 (48.9)	99 (69.7)	145 (45.6)	
Trader	15 (3.0)	6 (4.2)	9 (2.8)	
Full-time Parent	7 (1.4)	1 (0.7)	6 (1.9)	
Other	49 (9.8)	12 (8.5)	37 (11.6)	
Behaviors and Environment–n (%)				
Hunting in last 12 months	38 (7.6)	12 (7.5)	26 (7.7)	1
Fishing in the last 12 months	45 (9.0)	7 (4.3)	38 (11.3)	0.018*
Grazing in the last 12 months	257 (51.5)	63 (39.1)	194 (57.6)	<0.001*
Livestock in buildings	400 (80.2)	123 (76.9)	277 (82.7)	0.157
Wildlife near home	393 (78.8)	118 (73.3)	275 (81.4)	0.05*
Village Flooding	108 (21.6)	19 (11.8)	89 (26.3)	<0.001*
Village Drought	104 (20.8)	31 (19.3)	73 (21.6)	0.628
Water collected (wet season)–n (%)				
Pump	45 (9.0)	11 (6.8)	34 (10.1)	0.313
Roof Capture	499 (100.0)	161 (100.0)	338 (100.0)	NA
Tap	47 (9.4)	20 (12.4)	27 (8.0)	0.155
Spring	202 (40.5)	72 (44.7)	130 (38.5)	0.217
Well	67 (13.4)	29 (18.0)	38 (11.2)	0.05*
River	107 (21.4)	29 (18.0)	78 (23.1)	0.241
Dam	499 (100.0)	161 (100.0)	338 (100.0)	NA
Borehole	175 (35.1)	56 (34.8)	119 (35.2)	1
Water collected (dry season)—n (%)				
Pump	48 (9.6)	12 (7.5)	36 (10.7)	0.332
Roof Capture	499 (100.0)	161 (100.0)	338 (100.0)	NA
Tap	36 (7.2)	14 (8.7)	22 (6.5)	0.485
Spring	200 (40.1)	70 (43.5)	130 (38.5)	0.331
Well	60 (12.0)	25 (15.5)	35 (10.4)	0.13
River	124 (24.8)	32 (19.9)	92 (27.2)	0.096
Dam	499 (100.0)	161 (100.0)	338 (100.0)	NA
Borehole	177 (35.5)	57 (35.4)	120 (35.5)	1
Yellow Fever Vaccination—n (%)				0.06
Yes	30 (6.0)	4 (2.5)	26 (7.7)	
No	407 (81.6)	138 (85.7)	269 (79.6)	
Don't know	62 (12.4)	19 (11.8)	43 (12.7)	
Smoking—n (%)	32 (6.4)	6 (3.8)	26 (7.8)	0.139

* indicates significant value

Variables selected by the lowest AIC glmmLasso analysis predictive of an outcome of either alphavirus or flavivirus infection included education level, specifically pre-school (as compared to none) (OR = 0.36, p = 0.014, CI_95_: 0.16–0.81), occupation status of student (as compared to having no occupation) (OR = 0.36, p = 0.008, CI_95_: 0.17–0.77), grazing in the last 12 months (OR = 2.19, p < 0.001. CI_95_: 1.38 to 3.45), recent village flooding (OR = 2.49, p = 0.005, CI_95_: 1.31–4.73), and recent fever (OR = 0.52, p = 0.0275, CI_95_: 0.29–0.93) (Table 2). Significant variables selected for education level and occupation may also be used as a proxy for age. Similarly, statistically significant variables selected by CV analysis by glmmLasso with increased odds of exposure to alphavirus or flavivirus infection included sex (male, OR = 0.47, p = 0.009, CI_95_: 0.27–0.83), grazing in the last 12 months (OR = 2.49, p < 0.001, CI_95_: 1.45–4.26), and recent village flooding (OR = 2.75, p = 0.005, CI_95_: 1.35–5.63).

**Table 2:** Variables selected for significance relative to arbovirus infection.

AIC				
Variable	Odds Ratio	Standard Error	p-value	95% Confidence Interval
Intercept	2.43	0.12	0	1.9–3.1
Education Level (ref = None)				
Pre-school	0.36	0.41	0.01*	0.16–0.81
Primary	1.26	0.37	0.54	0.61–2.6
Secondary and above	0.75	0.75	0.69	0.17–3.2
Occupation (ref = None)				
Full-time parent	1.39	1.15	0.78	0.14–13. 34
Trader	0.33	0.62	0.08	0.10–1.14
Student	0.36	0.38	0.008*	0.17–0.77
Other	0.73	0.39	0.43	0.34–1.60
Behaviors (ref = No)				
Hunting in the last 12 months	0	NA	NA	NA
Fishing in the last 12 months	0	NA	NA	NA
Grazing in the last 12 months	2.19	0.23	< 0.001*	1.38–3.45
Village Variables (ref = No)				
Recent flooding	2.49	0.33	0.005*	1.31–4.73
Recent drought	0	NA	NA	NA
Health: Recent fever (ref = No)	0.52	0.30	0.028*	0.29–0.93
CV				
Variable	Odds Ratio	Standard Error	p-value	95% Confidence Interval
Intercept	Inf	264000	0.99	
Sex (ref = Female)	0.47	0.29	0.009*	0.27–0.83
Education Level (ref = None)				
Pre-school	0.60	0.44	0.26	0.25–1.44
Primary	1.71	0.44	0.22	0.73–4.03
Secondary and above	0.73	0.92	0.74	0.12–4.44
Occupation (ref = None)				
Full time parent	1.30	1.25	0.83	0.11–15.1
Trader	0.26	0.75	0.07	0.06–1.10
Student	0.39	0.81	0.25	0.08–1.91
Other	1.15	0.54	0.79	0.40–3.29
Animal Exposure (ref = No)				
Livestock in building	2.23	0.68	0.24	0.59–8.43
Poultry in building	0.79	0.65	0.71	0.22–2.83
Wildlife near home	1.88	0.35	0.07	0.94–3.75
Rats near home	0.93	0.42	0.85	0.41–2.09
Water Collected (ref = No)				
Wet season–Tap	0.61	0.80	0.54	0.12–2.95
Wet season–Well	0.52	1.05	0.53	0.07–4.01
Wet season–River	0.21	1.15	0.18	0.02–2.01
Dry season–Tap	0.61	0.92	0.59	0.10–3.69
Dry season–Well	1.11	1.09	0.92	0.13–9.50
Dry season—River	7.52	1.13	0.07	0.82–68.6
Behaviors (ref = No)				
Fishing in the last 12 months	2.01	0.54	0.19	0.70–5.77
Grazing in the last 12 months	2.49	0.27	< 0.001*	1.45–4.26
Village Variables (ref = No)				
Recent Flooding	2.75	0.36	0.005*	1.34–5.63
Health (ref = No)				
Recent illness	5.50	0.42	0.15	0.24–1.24
Recent fever	0.54	0.35	0.07	0.27–1.06
Yellow Fever Vaccination				
Yes	1.79	0.86	0.49	0.33–9.56
No	1.49	0.37	0.28	0.72–3.09

Binomial logistic regression analysis using glmmLasso chosen by the lowest AIC or CV value (n = 499).

* indicates significant value.

**Fig 1:**
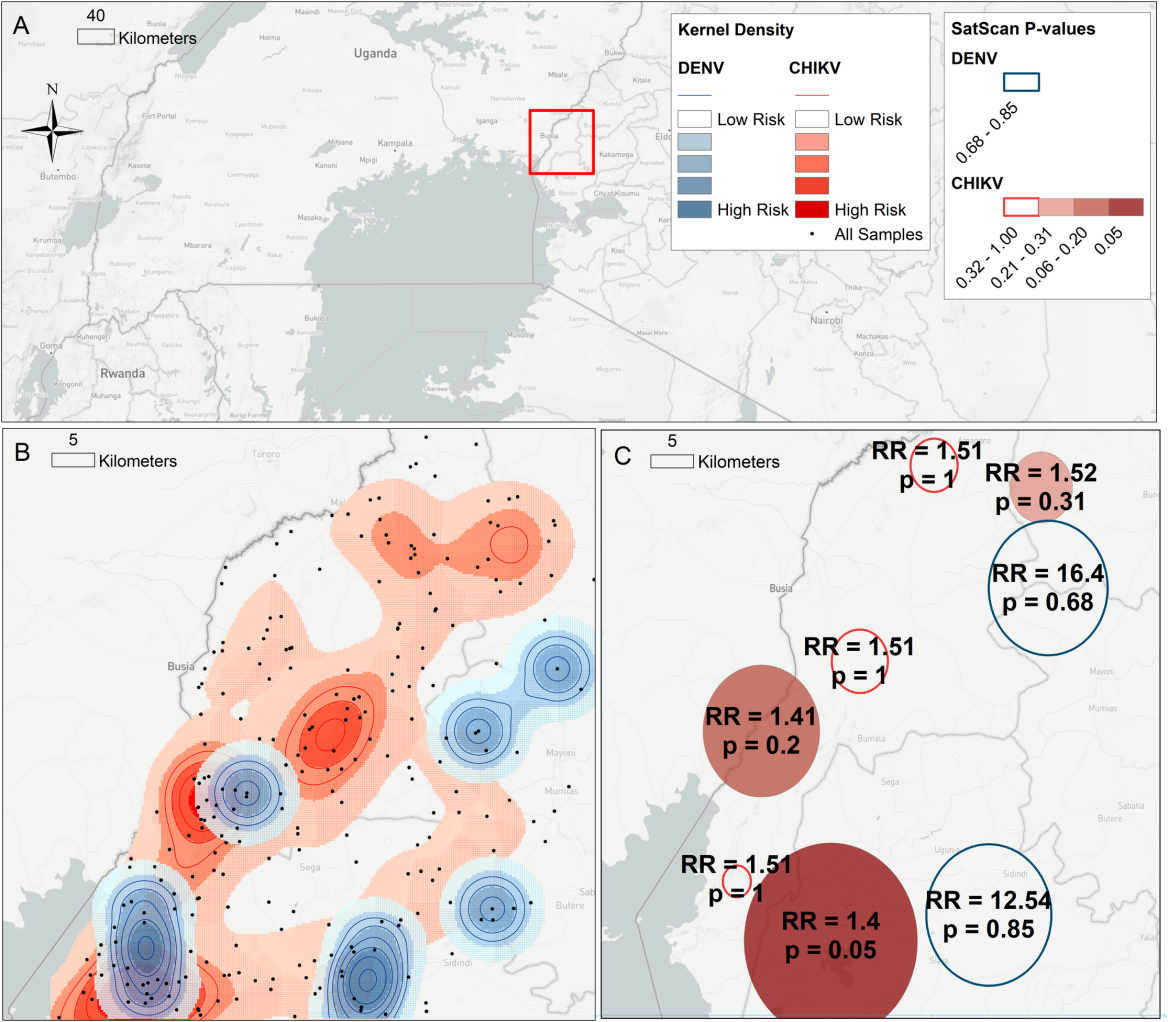
Kernel density and relative risk maps. Study location in western Kenya (a). Kernel density analysis of alphavirus, indicated by the presence of anti-CHIKV IgG, and flavivirus, indicated by anti-DENV IgG, cases in the study region (b). SatScan analysis showing RR of primary cluster compared to non-cluster with associated p-value (c). Infection and water bodies are also drawn (c).

Kernel density analysis was performed to examine spatial variation in the distribution of alphavirus or flavivirus exposure in the study area (Fig 1A and 1B). To quantify the spatial clustering, a spatial scan was performed to identify geographic clusters of higher than expected case counts in the distribution of alphavirus or flavivirus exposure in the study area (Fig 1C). Two clusters of flavivirus exposure were identified but were not statistically significant (Relative Risk (RR) inside cluster as compared to outside; RR = 12.5; p > 0.5 and RR = 16.4; p > 0.5). Strong evidence for spatial autocorrelation was identified for individual risk of alphavirus exposure with six clusters (RR range 1.4–1.5), and one statistically significant cluster (RR = 1.4; p = 0.05). This alphavirus exposure cluster was identified in the south-west corner of the study region, proximate to the Lakes Victoria, Kenyaboli, and Sare, and wetlands. Distance to nearest water body was associated with infection exposure (median distance = 1.2 km; IQR = 0.3–2.4) compared to non-infected (median = 1.7 km; IQR = 0.8–3.2) (Wilcoxon rank-sum p-value = 0.004).

## Discussion

Alphavirus seroprevalence was significantly higher than that of flavivirus, which is consistent with previous studies conducted in Busia[22]. Rates have not changed substantially in recent years, despite documented CHIKV and DENV outbreaks. A similar study by Mease et al. reported 59.91% seroprevalence for CHIKV, and 1.96% seroprevalence for DENV in 2004[22].

Flavivirus and alphavirus prevalence in Kenya is highly variable between regions. The low exposure to flaviviruses reported in our study is similar to that of previous surveys conducted within Busia[3] and other western parts of Kenya[20, 21]. However, other studies have found low CHIKV seroprevalence in Western Province[20]. Flavivirus exposure is much more likely in southern[46] and coastal Kenya[3, 21]. In a study by Sutherland et al. in 2011, 20% of inland subjects and 37% of coastal subjects tested positive for CHIKV by IgG ELISA[21]. A 2009 study by LaBeaud et al. reported prevalence of 26% for alphaviruses along coastal Kenya[2]. The regional variability of seroprevalence for flaviviruses and alphaviruses throughout Kenya suggests exposure is likely dependent on fluctuations in climate and weather patterns, environmental features, vector abundance, and mobility and access to transportation. Seroprevalence may also be linked to areas with a higher percentage of rural versus urban villages. However, risk factors for exposure to arboviruses are not reported in all of the studies previously conducted in Kenya, making it difficult to compare factors associated with exposure for each study population. Our study represents a rural, predominantly poor population that keeps small numbers of livestock on an individual homestead, and works outside during the day.

Experience of recent fever was found to be significantly relevant to IgG positives for either alphavirus or flavivirus infection, described as “arbovirus infection”, by AIC models, suggesting ongoing, interepidemic transmission of alphaviruses and flaviviruses in western Kenya. Adults aged ≥ 45 showed no exposure to flaviviruses by DENV IgG ELISA, suggesting either a more recent emergence of flaviviruses in this area, or a lower risk due to advanced age or behavioral factors. This may suggest exposure may vary by location, as students may be exposed at school, as opposed to in the homestead. The youngest seropositive participant was aged 5, which alludes to ongoing transmission of flaviviruses, despite lack of reported outbreaks in this region. The minimal number of flavivirus seropositives overall do not indicate flaviviruses as an emergent threat to the Busia region. Other regions of Kenya, such as central and coastal villages, have reported outbreaks of flaviviruses, resulting in high seroprevalence and risk for future outbreaks[2, 20]. Marginal flavivirus prevalence may be due to vector behavior, yet the extensive alphavirus prevalence suggests a regular, strong vector presence.

Our data suggest very early and common exposure to alphavirus infections in western Kenya. The youngest seropositive participant was 5 years, demonstrating ongoing and persistent exposure throughout life in this region, or persistent antibody from an early infection in childhood. Of note, older children were more likely to be seropositive than adults, which suggests more recent exposure to alphaviruses in this region within the last decade. The CV test model suggested females were at greater risk of exposure, indicating further investigation should be dedicated to arboviral disease disparities between males and females, especially relating to behavioral or occupational differences defined by gender roles defined in communities.

Kernel density and SatScan analysis of seropositives illustrated spatial clustering in the distribution of exposure to alphaviruses and flaviviruses in the study region. Areas of higher relative exposure to alphavirus and flavivirus transmission overlapped, with exposure appearing to cluster around regions with direct access to Lake Victoria. Regions such as Teso district, Bungoma county, and northern areas of Busia, which only showed high risk for alphaviruses (represented by CHIKV in Fig 1), are more than 100km away from Lake Victoria. Regions associated with high risk for both alphaviruses and flaviviruses were located primarily in households within close proximity to Lake Victoria, in the southwestern area of the study site. Proximity to bodies of water, such as Lakes, wetlands, or larger rivers, were also found to be associated with seroprevalence. Proximity to bodies of water may be related to occupation or homestead behaviors and activities, as fishing within the last 12 months was found to be significantly correlated with previous infection. Ecological variations throughout Kenya influence overall presence and abundance of vector species, as well as species diversity within specific environments[12, 24]. This contributes to variation in risk for specific arboviruses based on the presence and abundance of the vector. Regions within close proximity to Lake Victoria and smaller bodies of water are susceptible to flooding during wet seasons, which may increase potential environments for mosquito breeding. Studies in Thailand have shown populations of vector eggs increase exponentially between the beginning to the peak of rainy seasons, directly affecting arbovirus transmission[47]. Many believe flooding is an indicator of infection risk that results from fluctuating climate extremes, such as periods of prolonged drought followed by heavy rainfall[48–51], lead to rewetting of environments and increased water pooling and innocuous water collection around homesteads. Our results show that flooding is significantly correlated to infection, whereas drought is not, contributing further evidence for the importance of rewetting and subsequent flooding as a result of heavy rainfall in arbovirus transmission. Regions with differential exposure to alpha- or flavivirus transmission are of interest for future risk analyses.

Grazing was consistently found to be significant in univariate and multivariate analyses. Activities involving animal exposure are not typically considered risk factors for alphaviruses such as CHIKV, and flaviviruses such as DENV, as transmission in humans is limited to mosquito bite. However, as climate and environment fluctuate between drought and flooding, individuals herding and grazing livestock may have an increased risk of mosquito exposure as breeding habitats expand[12]. Variables describing recent hunting, fishing, and grazing behaviors may act as proxies for time spent outdoors, where *Aedes aegypti* breeding sites are more common. It is also likely that factors such as time outdoors and travel to or through high transmission areas that are not located near the homestead, through grazing, fishing, and herding activities, may increase risk of exposure to alphaviruses and flaviviruses.

Overlapping areas of high exposure may be due to behavior of the vector that is shared between alphaviruses and flaviviruses[24], due to environmental factors that support mosquito proliferation, or behavioral practices that impact risk of exposure to arboviral diseases. Given the extremely low number of participants seropositive for previous flavivirus exposure, we believe the overlapping seropositives for both alphaviruses and flaviviruses could provide evidence of saturation of alphavirus exposure in this area. Co-infection of specifically CHIKV and DENV have been reported in mosquitoes and humans during a number of overlapping outbreaks[23, 52–54], and in mosquitoes via artificial oral exposure in a laboratory setting[55]. Yet, others suggest that competitive suppression occurs when cultured mosquito cells are co-infected with CHIKV and DENV[56], which may explain the regional variability of alphavirus and flavivirus prevalence that has been continually reported throughout Kenya[2, 20–22].

No outbreaks of alphaviruses have been described in this region in the last 10 years, despite common exposure. It is likely that some of the exposure represented here did not manifest clinically; however, any participants who suffered clinical disease, likely did not garner a diagnosis of arboviral infection. The clinical presentations of arboviral diseases are often highly non-specific, with the exception of severe and persistent arthralgia associated with CHIKV infections experienced by 7–79% of patients [16]. The non-specific, febrile clinical presentation of arboviruses is commonly indistinguishable from each other and from malaria[19], which may have an effect on the accuracy of diagnoses and regional incidence reports. There are currently no diagnostics for arboviruses being routinely implemented in health centers in the study area[57], therefore clinical cases would rarely be identified outside of research studies or referral hospitals outside of the region. Additionally, persons experiencing non-specific or mild symptoms may not seek medical attention, whether due to low-impact illness, or to other boundaries that can restrict access to medical care, such as cost, limited access to transportation, distance required to travel to a medical facility, occupational or childcare responsibilities, or corruption, which may cause inaccuracies or biases in the estimation of alphavirus and flavivirus burden in many areas.

There are some limitations of this study that should be considered. All samples were tested for previous exposure to alphaviruses and flaviviruses by indirect IgG ELISA against CHIKV and DENV_1–4_, which has limited specificity against cross-reactivity within each viral genus[58–60]. Viral specificity can be identified by PRNTs, which were not performed. The questionnaire utilized during sample collection was not designed to test the hypothesis, which may explain why so few variables were chosen by the glmmLasso analysis. Questionnaires did not include questions about regular exposure to mosquitoes, homestead structures that may support mosquito breeding or access, or mosquito abatement behaviors, whether on an individual or community level, which limits our ability to assess the risk factors directly related to vector behavior and exposure. Individual-level mosquito exposure is a specific area that deserves a more detailed investigation in order to fully understand the risks of arboviral transmission in the study region. Mosquito behavior was not available for integration into the kernel density analysis, which also limited our ability to definitively link mosquito abundance and likelihood of exposure to positive event densities. Due to low number of cases identified by this study, we could not present odds ratio by sub-region. Neither was the population density available at a finer scale to support estimates adjusted for population density. The cases represent a random sample of a weighted and stratified random sampling of households in the study area. The sampling and thus distribution of cases may over-represent geographic regions with greater cattle populations as a result of the sampling strategy.

Our findings indicate minimal flavivirus exposure and significant alphavirus exposure in the Busia region of western Kenya. Despite our intentions of surveying prevalence and prior exposure, experience of recent fever was found to be significant to arbovirus infection, suggesting recent exposure and acute disease, and possible interepidemic transmission in this area of Kenya. Alphavirus exposure is common and occurs early on in childhood, which may have important, yet undetermined health implications. The high prevalence of alphavirus reported here, in combination with the extensive spatial clusters of autocorrelation with alphavirus exposure, indicates that vector populations are consistently prolific in western Kenya, suggesting that there are other factors influencing DENV exposure. Kernel density analysis results indicate overlapping regions of exposure for alphavirus and flavivirus transmission in western Kenya, especially in homesteads located close to Lake Victoria, suggesting environmental, behavioral, or demographic factors influence differential exposure, despite transmission by the same vectors. Further research is required to accurately determine the burden and impact of arboviruses in different localities. There is a need to increase surveillance for these infections amongst patients presenting with fever in health facilities. The presence of arboviruses in Kenya is undisputable, yet the prevalence data currently available does not accurately represent the severity of exposure, infection, and disease as it varies by region.

## Supporting information

S1 Document Strobe checklist for cross-sectional studies. (DOC)
