# Supplementary material for: Serological and spatial analysis of alphavirus and flavivirus prevalence and risk factors in a rural community in western Kenya
Source: PLoS Negl Trop Dis. 2017 Oct 17;11(10):e0005998. doi: 10.1371/journal.pntd.0005998 (PMC5659799; doi:10.1371/journal.pntd.0005998)
Supplement: S1 Document — (DOC) [file pntd.0005998.s001.doc]

**Submission PNTD-D-17-01006R1**

STROBE Statement—Checklist of items that should be included in reports of ***cross-sectional studies***

|  | Item No | Recommendation | Author Checklist & Notes |
| --- | --- | --- | --- |
| **Title and abstract** | 1 | (*a*) Indicate the study’s design with a commonly used term in the title or the abstract | **✓Title indicates serological and spatial analysis was conducted on individuals from a rural community in western Kenya** |
|  |  | (*b*) Provide in the abstract an informative and balanced summary of what was done and what was found | **✓Abstract describes summary of research and findings** |
| Introduction | | |  |
| Background/rationale | 2 | Explain the scientific background and rationale for the investigation being reported | **✓ First two paragraphs of the introduction provide background information, third paragraph explains rationale of the specific study** |
| Objectives | 3 | State specific objectives, including any prespecified hypotheses | **✓ Final sentence of the introduction states “In this study, we aimed to increase the knowledge regarding seroprevalence and factors associated with increased exposure to alphaviruses and flaviviruses in a population of children and adults living in western Kenya.”** |
| Methods | | |  |
| Study design | 4 | Present key elements of study design early in the paper | **✓ See Methods section with subsections (starts on page 4, line 88)** |
| Setting | 5 | Describe the setting, locations, and relevant dates, including periods of recruitment, exposure, follow-up, and data collection | **✓ See “study area” of methods section (page 4, lines 90-94) and “Sample population” of methods section (pages 4-5, lines 95-116)** |
| Participants | 6 | (*a*) Give the eligibility criteria, and the sources and methods of selection of participants | **✓ See “Sample population” subsection of Methods section, specifically lines 107-115)** |
| Variables | 7 | Clearly define all outcomes, exposures, predictors, potential confounders, and effect modifiers. Give diagnostic criteria, if applicable | **✓ See data analysis section (pages 6-7 , lines 135 – 184), specifically lines regarding variable selection via statistical models glmmLasso, AIC, BIC, and CV criteria. Diagnostic criteria is described in the “serological analysis section (page 5, lines 117-133).** |
| Data sources/ measurement | 8* | For each variable of interest, give sources of data and details of methods of assessment (measurement). Describe comparability of assessment methods if there is more than one group | **✓ See data analysis section (pages 6-7 , lines 135 – 184), specifically lines regarding variable selection via statistical models glmmLasso, AIC, BIC, and CV criteria.** |
| Bias | 9 | Describe any efforts to address potential sources of bias | **✓ See “sample population” subsection (page 4, lines 99-100, and lines 110 -111 regarding randomization of sample population), data analysis section (pages 6-7 , lines 135 – 184), specifically lines regarding randomization effect. Weighted and stratified random sampling of homesteads is also referenced in the Ethics statement section (page 7-8, lines 186 – 193).** |
| Study size | 10 | Explain how the study size was arrived at | **✓ See “sample population” subsection (page 4-5, lines 107 – 111).** |
| Quantitative variables | 11 | Explain how quantitative variables were handled in the analyses. If applicable, describe which groupings were chosen and why | **✓ Questionnaires were used for each participant (see pg 5, lines 111-114) to collect relevant demographic and health information, in combination with a one-time blood collection (page 4, lines 105-106). Variable selection is described in data analysis section “pages 6-7, lines 135 – 184), and again in the Results section.** |
| Statistical methods | 12 | (*a*) Describe all statistical methods, including those used to control for confounding | **✓ See data analysis section (pages 6-7 , lines 135 – 184), specifically lines regarding variable selection via statistical models glmmLasso, AIC, BIC, and CV criteria. Kernal and spatial analysis is also described in this section.** |
|  |  | (*b*) Describe any methods used to examine subgroups and interactions | **✓ See data analysis section (pages 6-7 , lines 135 – 184), specifically lines regarding variable selection via statistical models glmmLasso, AIC, BIC, and CV criteria. Kernal and spatial analysis, also described in this section, was used to identify spatial interactions between exposure regions.** |
|  |  | (*c*) Explain how missing data were addressed | **Any missing data was not included in analyses, unless otherwise denoted as a “none” response.** |
|  |  | (*d*) If applicable, describe analytical methods taking account of sampling strategy | **✓ See data analysis section (pages 6-7 , lines 135 – 184), specifically lines regarding variable selection via statistical models glmmLasso, AIC, BIC, and CV criteria.** |
|  |  | (*e*) Describe any sensitivity analyses | **✓ See data analysis section (pages 6-7 , lines 135 – 184), specifically lines regarding variable selection via statistical models glmmLasso, AIC, BIC, and CV criteria. Kernal and spatial analysis sensitivity is also described in this section.** |
| Results | | |  |
| Participants | 13* | (a) Report numbers of individuals at each stage of study—eg numbers potentially eligible, examined for eligibility, confirmed eligible, included in the study, completing follow-up, and analysed | **✓All of this information is included in the sample population selection, as one time sampling was used, and samples were maintained via biobanking. No follow-ups required for this project. Basic summarization of overall findings is described in the Results section (page 8, lines 196-204).** |
|  |  | (b) Give reasons for non-participation at each stage | **N/A, one time sampling.** |
|  |  | (c) Consider use of a flow diagram | **N/A, one time sampling.** |
| Descriptive data | 14* | (a) Give characteristics of study participants (eg demographic, clinical, social) and information on exposures and potential confounders | **✓ Basic summarization of overall findings is described in the Results section (page 8, lines 196-204). Specifically significant variables are discussed in following paragraphs in the discussion section.** |
|  |  | (b) Indicate number of participants with missing data for each variable of interest | **Any missing data was not included in analyses, unless otherwise denoted as a “none” response.** |
| Outcome data | 15* | Report numbers of outcome events or summary measures | **✓ See tables 1 and 2** |
| Main results | 16 | (*a*) Give unadjusted estimates and, if applicable, confounder-adjusted estimates and their precision (eg, 95% confidence interval). Make clear which confounders were adjusted for and why they were included | **✓ See tables 1 and 2** |
|  |  | (*b*) Report category boundaries when continuous variables were categorized | **✓ See tables 1 and 2** |
|  |  | (*c*) If relevant, consider translating estimates of relative risk into absolute risk for a meaningful time period |  |
| Other analyses | 17 | Report other analyses done—eg analyses of subgroups and interactions, and sensitivity analyses | **✓ See Figures 1a-c** |
| Discussion | | |  |
| Key results | 18 | Summarise key results with reference to study objectives | **✓ See summary paragraph (page 17, lines 375-389) for concise summarization. Each significant variable is discussed in subsequent discussion section paragraphs.** |
| Limitations | 19 | Discuss limitations of the study, taking into account sources of potential bias or imprecision. Discuss both direction and magnitude of any potential bias | **✓ See limitations paragraph (page 16-17, lines 360-374).** |
| Interpretation | 20 | Give a cautious overall interpretation of results considering objectives, limitations, multiplicity of analyses, results from similar studies, and other relevant evidence | **✓ See limitations paragraph (page 16-17, lines 360-374).** |
| Generalisability | 21 | Discuss the generalisability (external validity) of the study results | **✓ See summary paragraph (page 17, lines 375-389).** |
| Other information | | |  |
| Funding | 22 | Give the source of funding and the role of the funders for the present study and, if applicable, for the original study on which the present article is based | **✓See financial disclosure in manuscript submission form.** |

*Give information separately for exposed and unexposed groups.

**Note:** An Explanation and Elaboration article discusses each checklist item and gives methodological background and published examples of transparent reporting. The STROBE checklist is best used in conjunction with this article (freely available on the Web sites of PLoS Medicine at http://www.plosmedicine.org/, Annals of Internal Medicine at http://www.annals.org/, and Epidemiology at http://www.epidem.com/). Information on the STROBE Initiative is available at www.strobe-statement.org.
